# Gastrointestinal perforations in patients with rheumatoid arthritis treated with biological disease-modifying antirheumatic drugs in Sweden: a nationwide cohort study

**DOI:** 10.1136/rmdopen-2020-001201

**Published:** 2020-07-14

**Authors:** Andrei Barbulescu, Bénédicte Delcoigne, Johan Askling, Thomas Frisell

**Affiliations:** Department of Medicine Solna, Karolinska Institutet, Stockholm, Sweden

**Keywords:** Rheumatoid Arthritis, Treatment, DMARDs (biologic), Epidemiology

## Abstract

**Objectives:**

To compare incidence rates of gastrointestinal (GI) perforations between patients with RA and the general population, and between patients treated with tumour necrosis factor inhibitors (TNFi) and non-TNFi biologics.

**Methods:**

In this nationwide cohort study, a total of 63 532 patients with RA, with 26 050 biological treatment episodes (TNFi, rituximab, abatacept or tocilizumab) and 76 304 general population controls, were followed between 2009 and 2017 until the first outcome event. The main outcome was hospitalisation or death due to lower GI perforations, identified according to a prespecified list of ICD-10 (International Classification of Diseases, 10th revision) codes. Inverse probability of treatment weighting was used for adjustment.

**Results:**

The sex-standardised and age-standardised incidence rates of lower GI perforations were 1.1 (95% CI 1.0 to 1.3) events per 1000 person-years among general population controls, 1.6 (1.5–1.7) among bionaïve patients and ranged from 1.8 (1.4–3.6) (TNFi) to 4.5 (2.7–10.4) (tocilizumab) among biologics-treated patients. After adjustment for glucocorticoid use, the risk in bionaïve, TNFi-treated, abatacept-treated or rituximab-treated patients with RA was no longer different from the general population, while for tocilizumab it remained significantly higher. Comparing tocilizumab to TNFi, the adjusted HR for lower GI perforations was 2.2 (1.3–3.8), corresponding to one additional GI perforation per 451 patient-years treated with tocilizumab instead of TNFi.

**Conclusion:**

Tocilizumab was associated with a higher risk of lower GI perforations compared with alternative biologics. In absolute numbers, the risk remained low on all biologics commonly used to treat RA, but the accumulated evidence across settings and outcome definitions supports that this risk should be considered in treatment guidelines for RA.

## INTRODUCTION

Patients with rheumatoid arthritis (RA) have been reported to be at an increased risk of gastrointestinal (GI) complications compared to patients who did not have RA.^1 2^ Among these, GI perforations are rare but potentially lethal, and it is unclear to what extent a risk increase may be caused by inflammation or other RA processes, or rather by the treatments used in RA.

An association to treatment is established for glucocorticoids (GC), which have been shown to increase the risk of bleeding and perforation in both upper^3^ and lower GI tract,^4^ and non-steroidal anti-inflammatory drugs (NSAIDs), where recent evidence suggest involvement in intestinal perforation in addition to well-known risk increases for upper GI ulceration.^5^

While conventional synthetic disease-modifying antirheumatic drugs (csDMARDs) do not seem to modify the risk of GI perforations,^6^ uncertainty remains around the possible risks associated with biological disease-modifying antirheumatic drugs (bDMARDs). Cases of GI perforations have been reported in etanercept-treated patients,^7^ and GI perforations are included in the product information of infliximab, adalimumab and certolizumab as uncommon or rare adverse reactions.^8–10^ However, one large analysis of data from the British Society for Rheumatology Biologics Register found no difference in risk, comparing tumour necrosis factor inhibitor (TNFi) versus csDMARDs, after adjusting for GC use and other risk factors.^11^

Data on other non-TNFi bDMARDs and comparisons between bDMARDs is limited,^12^ but available data suggests a possibly increased risk of lower GI perforations associated with tocilizumab. A safety signal was first triggered by several cases reported in preapproval clinical trials and postmarketing surveillance for tocilizumab.^13–15^ However, clinical trials were not designed to study this rare outcome, providing insufficient follow-up time, especially in the comparator arm.^13^ Also, postmarketing surveillance via spontaneous adverse drug reaction reports is prone to reporting bias due to perceptions about risks. Strangfeld *et al* analysed data from RABBIT (the German biologics register) and found tocilizumab to be associated with a more than fourfold increase in the risk of lower GI perforations compared to csDMARDs.^16^ No other bDMARD was associated with an increased risk. However, the study had limited ability to adjust for baseline disease history. Two studies analysed US claims data,^14 17^ comparing abatacept, rituximab and tocilizumab with TNFi. The reported risk of lower GI perforations was again significantly higher for tocilizumab but not for the other non-TNFi bDMARDs. However, the data sources used in these studies lacked measures of, and the analyses could thus not be adjusted for, RA disease activity or inflammation markers, which at least in Sweden is known to influence the choice of bDMARD.^18^

With differences in outcome definitions, limited statistical power and lack of data on important confounders in these previous studies, additional evidence is warranted before strong conclusions on the possibly increased risk of GI perforations in RA, and specifically with tocilizumab, can be drawn. Therefore, we aimed to estimate and compare incidence rates of GI perforations among Swedish patients with RA using different treatments, and to a matched general population comparator, with particular focus on TNFi versus non-TNFi bDMARDs.

## PATIENTS AND METHODS

### Study design

We conducted a nationwide, register-based cohort study, comparing the incidence of GI perforations between patients with RA and a general population sample, and between different bDMARDs used in RA.

### Data sources

Several Swedish national registers served as data sources for this study. Inpatient and specialty outpatient visit records were identified in the National Patient Register.^19^ Cancer diagnoses were identified in the Swedish Cancer Register. RA parameters and treatments were identified in the Swedish Rheumatology Quality Register (SRQ) that collects longitudinal data from rheumatology visits. The Swedish Biologics Register (ARTIS), that is part of the SRQ, covers more than 90% of biological treatments in RA from their introduction to clinical practice in Sweden (1999), and was used as data source for bDMARD treatments.^20 21^ Dispensed outpatient prescriptions were identified in the Prescribed Drugs Register, with virtually complete coverage since July 2005.^22^ Demographic data and data on emigration were extracted from the Total Population Register^23^ while death date and causes were extracted from the Swedish Cause of Death Register.^24^ The linkage between national registers was realised by identifying individuals using their unique personal identification number.^25^

### Settings and patients

Patients with RA were identified in the National Patient Register as having minimum two recorded ICD-10 (International Classification of Diseases, 10th revision) diagnoses of *M05* or *M06*, at least one from a rheumatology or internal medicine specialist.^26^ Starting from 2009, all bDMARDs under study were available for clinical use, thus all bDMARD treatment starts after January 2009 and were extracted from SRQ/ARTIS. General population controls were matched 5:1 by sex, age and geographical location to the biologic-treated patients. All patients were followed from the earliest January 1, 2009 to the latest December 31, 2017 (the end of data availability).

### Outcome

Hospitalisations with a main or secondary diagnosis of GI perforation and death due to GI perforation (primary or contributory cause of death) were counted as outcome events. Outcome definitions with ICD-10 and procedure codes are summarised in online supplementary table 1. Lower GI perforations were considered the main outcome, as they made up 85% of all perforations (online supplementary table 2), with all GI perforations assessed in supplementary analyses.

10.1136/rmdopen-2020-001201.supp1 Supplementary data

### Exposure and follow-up

Follow-up of general population controls started at the same date as the first biological treatment of the matched patient with RA and was censored if the control developed RA. Patients were followed as bionaïve from first fulfilment of the RA diagnostic criteria to first start of a biological treatment, as identified in SRQ. If RA criteria had been fulfilled before the start of the study period, bionaïve follow-up would start on January 1, 2009. Patients starting a bDMARD before the start of the study period were not included in the bionaïve cohort. Biologic-treated patients were followed in each treatment cohort from the date of treatment start, to the date of decision to end treatment or initiation of a new biologic, plus an extension period of 90 days (intended to capture events recorded after a decision to stop therapy due to early symptoms). Follow-up under any exposure cohort was censored at death, emigration, end of data or at first outcome event. The following biologics were studied: TNFi (etanercept, infliximab, adalimumab, certolizumab pegol and golimumab) as one group, rituximab, abatacept and tocilizumab, regardless of formulation or route of administration. Patients could contribute follow-up to several exposure cohorts, but only the first treatment with each bDMARD was included. Thus, patients who switched bDMARDs during the study period would contribute to repeated observations, under different drugs, and could also contribute to several outcome events, if they repeatedly experienced outcome events under different treatments.

### Missing data

Data was complete for most variables derived from national registers, including the outcome. Nevertheless, missing data was present for education level and for several variables extracted from SRQ representing disease activity or co-medication, used for confounding adjustment in the comparison between bDMARD treatments. The proportion of missing data for each variable is presented in online supplementary table 3. In order to use the entire outcome data and reduce selection bias due to analysing only complete observations, we used multiple imputation with fully conditional specification.^27^ A comparison between the distribution of observed data before imputation and complete data after imputation is presented in online supplementary table 3. The analysis dataset was imputed 20 times, with 25 burn-in iterations. Imputation models included only the analytical variables without interaction terms. Continuous variables were parametrised as cubic polynomials, except the baseline cumulated exposure to GCs and NSAIDs and number of hospitalisations, which were categorised into quartiles. As recommended in survival analysis using multiple imputation, the outcome was included as event/censoring binary indicator together with the Nelson-Aalen cumulative baseline hazard estimate.^28^ Each of the 20 imputed datasets were analysed separately and then estimates were pooled using Rubin’s rules.^29^

### Statistical analysis

Incidence rates in patients with RA and general population controls were standardised to the sex and age (10-year groups) distribution of the entire study population, estimating CIs with the Fay and Feuer method.^30^ HRs comparing each RA cohort to the general population were estimated in a multivariable Cox regression adjusting for age, sex and cumulated use of GCs 1 year before baseline.

The comparison between bDMARDs was adjusted for a larger set of covariates, including RA-specific factors not possible to adjust for in a comparison involving the general population. Due to the low number of outcome events, confounding adjustment was done using inverse probability of treatment weighting (IPTW).^31^ Stabilised IPTWs were calculated, within each imputed dataset, for each subject, as the inverse of their probability to receive the treatment they had in fact received considering their baseline characteristics, predicted using multinomial logistic regression, multiplied by the sample proportion with that treatment.^32^ These IPTWs were used to adjust survival curves,^33^ HRs in Cox models and incidence rates in generalised estimating equation Poisson models (with independent working correlation). The robust sandwich estimator was used for calculating 95% CIs in all Cox regressions. This approach corrects the variance after clustering due to IPT weighting and repeated observations, providing slightly conservative CIs.^34–36^

Numbers needed to harm were calculated as the inverse of the differences in adjusted incidence rates.

The time scale used in all Cox regressions was time since start of follow-up under an exposure cohort.

Baseline characteristics were measured before each start of follow-up. Thus, patients who restart follow-up under different exposure cohorts would have their baseline information updated at each start. Baseline characteristics were considered for confounding adjustment if they were thought to influence the choice of treatment (or were consequences of factors that influence treatment choice) and were also risk factors for GI perforation. No selection was done based on statistical tests of association with exposure and outcome. Baseline characteristics included in the IPTW adjustment model were age, sex, education level and year at start of follow-up, history of GI perforations, diverticular disease, intestinal ischemia, inflammatory bowel disease, other GI diseases, diabetes, chronic obstructive pulmonary disease, hospitalised infections, cardiovascular disease, cancer, surgical interventions on joints and the number of hospitalisations within 5 years before baseline, Health Assessment Questionnaire (HAQ) score, erythrocyte sedimentation rate (ESR), C reactive protein (CRP), rheumatoid factor, DAS28-CRP, RA duration, GC and NSAID use (baseline use and cumulated exposure within 1 year before baseline), and baseline use of methotrexate, other csDMARDs and selective cyclooxygenase 2 (COX2) inhibitors. Details of covariate definitions are in online supplementary table 4. The cumulated exposure to GCs and NSAIDs was estimated by (1) extracting all prescriptions within 1 year before baseline for each patient, (2) dividing the collected quantity of drug (mg) on each prescription by the corresponding WHO ‘defined daily dose’ (mg/day)^37^ to obtain the number of defined daily doses (DDDs) and (3) for each observation (patient) summing up the number GC DDDs and the number of NSAID DDDs. The defined daily dose represents the quantity of drug used per day by an average patient for the main indication of the drug.^37^ Continuous variables were parametrised in the same way as in the imputation models.

The distribution of weights was evaluated within each imputation and over all imputations (online supplementary figure 1). To assess achieved balance of confounding distributions between treatments, expected values for each confounder (proportion or mean) are presented before and after weighting in online supplementary table 5.

Adding the line of biological therapy to the IPTW denominator model, in order to adjust for it by weighting, induces extreme weights and unstable estimates, because the line of biological therapy is strongly associated with the exposure variable.^38^ However, the line of therapy could be added to both numerator and denominator of IPTW and also to the outcome model. In this way, while other confounders are adjusted by weighting, line of therapy is adjusted by conditioning on it.^39^

All data management and analysis were conducted using SAS 9.4 (SAS Institute).

### Secondary analyses

Incidence rates of any (lower or upper) GI perforation and risk contrasts between cohorts are presented in the online supplementary material.

To assess comparability with previous reports, incidence rates of any GI perforation were calculated, mimicking outcome definitions used in previous studies.^14 17^ A specific definition excluded diagnosis codes for diverticulitis, diverticulosis or ischemic colitis. Another broader, more sensitive definition included these events, together with codes for several intestinal surgical procedures. ICD-9CM and Current Procedural Terminology codes were used in the original definitions. We translated these to ICD-10 and (Nordic Medico-Statistical Committee (NOMESCO) procedural codes used in Sweden. Our translation of these definitions is presented in online supplementary table 1, together with ICD-10 codes used in our own definition.

Mortality was calculated as the proportion of cases that were either identified directly in the Cause of Death Register or were followed by death either during hospitalisation or within 90 days after discharge. As the number of cases was small, Fisher’s exact test was used for testing that mortality was equally distributed between treatment cohorts.

Since experiencing a GI perforation previously is an important risk factor for a recurrent GI perforation, we adjusted for history of GI perforation in the main analysis. In a sensitivity analysis, we excluded any patients with a history of GI perforation. In this analysis, patients could participate with repeated observations but with no more than one event.

## RESULTS

### Study population

We included 76 304 general population controls, 62 532 bionaïve patients with RA, 17 594 initiations of TNFi, 2527 of abatacept, 3552 of rituximab and 2377 of tocilizumab. The number of follow-up episodes in each exposure group and baseline characteristics are displayed in table 1. Comparing bionaïve patients with RA with those starting bDMARDs, the bionaïve were on average older, less educated and less likely to be women. Joint surgery was less frequent among bionaïve patients, indicating less severe RA. The most frequently used bDMARDs were TNFi—(68% of all biological treatment episodes). Patients treated with TNFi had better overall health and were earlier in their RA disease, compared to those starting non-TNFi bDMARDs. They also received less GC comedication. Proportions of historical GI conditions (eg, diverticular disease, perforations) were higher in patients starting non-TNFi compared to those starting TNFi bDMARDs, although tocilizumab initiators had lower proportions than those starting abatacept or rituximab.

**Table 1:** Characteristics of study participants at baseline (start of each follow-up episode)

Characteristic median (IQR) or N (%)	Bionaïve	TNFi	Abatacept	Rituximab	Tocilizumab	General population
No. of follow-up episodes	62 532	17 594	2527	3552	2377	76 304
*Demographics*						
Women	44 066 (70.5)	13 320 (75.7)	2031 (80.4)	2692 (75.8)	1885 (79.3)	57 787 (75.7)
Age (years)	66 (56–76)	59 (47–67)	61 (51–69)	64 (54–72)	59 (49–67)	60 (49–68)
*Highest education*						
9 years or less	21 176 (35.4)	3912 (22.4)	606 (24.2)	960 (27.4)	540 (23.0)	15 698 (20.9)
10– 12 years	25 603 (42.8)	8293 (47.5)	1230 (49.2)	1643 (47.0)	1131 (48.1)	33 928 (45.1)
More than 12 years	13 014 (21.8)	5264 (30.1)	666 (26.6)	896 (25.6)	679 (28.9)	25 607 (34.0)
*Disease history* *						
GI perforations	396 (0.6)	80 (0.5)	29 (1.1)	31 (0.9)	14 (0.6)	233 (0.3)
Diverticular disease	1961 (3.1)	481 (2.7)	121 (4.8)	161 (4.5)	84 (3.5)	1385 (1.8)
Intestinal vascular disease	62 (0.1)	10 (0.1)	2 (0.1)	4 (0.1)	1 (0.0)	24 (0.0)
Other GI disorders	6353 (10.2)	1493 (8.5)	325 (12.9)	446 (12.6)	242 (10.2)	4090 (5.4)
IBD	698 (1.1)	235 (1.3)	37 (1.5)	40 (1.1)	32 (1.3)	697 (0.9)
Diabetes	5634 (9.0)	1181 (6.7)	246 (9.7)	351 (9.9)	182 (7.7)	3548 (4.6)
COPD	2967 (4.7)	416 (2.4)	128 (5.1)	180 (5.1)	77 (3.2)	1267 (1.7)
Hospitalised infections †	4016 (6.4)	520 (3.0)	216 (8.5)	298 (8.4)	118 (5.0)	1037 (1.4)
Cardiovascular disease	21 197 (33.9)	3938 (22.4)	820 (32.4)	1247 (35.1)	643 (27.1)	11 875 (15.6)
Cancer ‡	7131 (11.4)	923 (5.2)	185 (7.3)	522 (14.7)	139 (5.8)	6262 (8.2)
Joint surgery	7071 (11.3)	2529 (14.4)	534 (21.1)	825 (23.2)	491 (20.7)	2431 (3.2)
No. of hospitalisations	1.0 (0.0–2.0)	0.0 (0.0–2.0)	1.0 (0.0–3.0)	1.0 (0.0–3.0)	1.0 (0.0–2.0)	0.0 (0.0–1.0)
*RA parameters*						
Disease duration (years)	‒	8.6 (3.3–17.3)	11.5 (5.5–20.6)	12.5 (5.7–21.7)	10.5 (4.7–19.2)	‒
Rheumatoid factor	‒	12 957 (75.9)	1953 (79.2)	3044 (87.5)	1829 (78.7)	‒
ESR	‒	18.0 (9.0–34.0)	22.0 (10.0–40.0)	27.0 (14.0–45.0)	26.0 (13.0–47.0)	‒
CRP	‒	7.0 (3.0–18.0)	8.0 (3.0–21.0)	10.0 (4.8–27.0)	11.0 (5.0–29.0)	‒
DAS28CRP score	‒	4.4 (3.5–5.2)	4.6 (3.9–5.3)	4.7 (3.8–5.4)	4.8 (4.0–5.6)	‒
HAQ score	‒	1.0 (0.6–1.5)	1.3 (0.9–1.8)	1.3 (0.9–1.8)	1.3 (0.9–1.8)	‒
*Comedication*						
Methotrexate	‒	9299 (65.7)	1163 (55.8)	1648 (56.3)	1030 (50.5)	‒
Other csDMARDs	‒	1887 (13.3)	215 (10.3)	441 (15.1)	203 (10.0)	‒
Selective COX2 inhibitors	‒	366 (2.6)	54 (2.6)	66 (2.3)	60 (2.9)	‒
Other NSAIDs	‒	4427 (31.3)	690 (33.1)	941 (32.2)	740 (36.3)	‒
Glucocorticoids	‒	6568 (46.4)	1180 (56.6)	1688 (57.7)	1109 (54.4)	‒
*Medication history (cumulated dispensed quantity)* †						
NSAIDs	33.3 (0.0–183.3)	66.7 (0.0–266.7)	65.0 (0.0–266.7)	50.0 (0.0–266.7)	100.0 (0.0–300.0)	0 (0.0–0.0)
Glucocorticoids	50.0 (0.0–150.0)	75.0 (0.0–200.0)	112.5 (25.0–225.0)	126.7 (28.1–250.0)	100.0 (10.0–225.0)	0 (0.0–0.0)

*Within the last 5 years before episode start (except cancer and hospitalised infections).

†Within 1 year before episode start.

‡Ever recorded before episode start.

COPD, chronic obstructive pulmonary disease; COX, cyclo-oxygenase; CRP, C reactive protein; csDMARD, conventional synthetic disease-modifying antirheumatic drug; DAS28CRP, Disease Activity Score using 28 joints and CRP; ESR, erythrocyte sedimentation rate; GI, gastrointestinal; HAQ, Health Assessment Questionnaire; IBD, inflammatory bowel disease; NSAID, non-steroidal anti-inflammatory drug; RA, rheumatoid arthritis; TNFi, tumour necrosis factor inhibitor.

### Follow-up time

**Figure 1:**
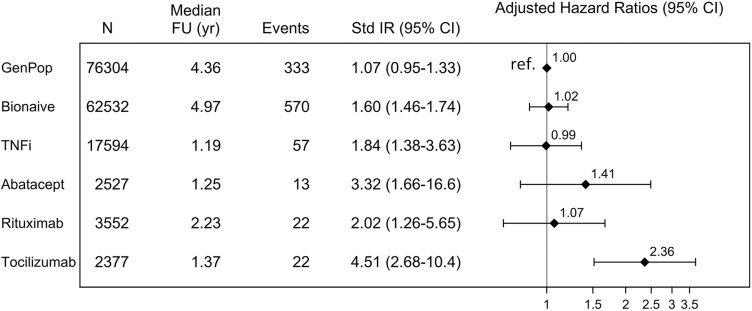
Lower gastrointestinal perforation incidence rates and contrast between patients with rheumatoid arthritis and the general population. *Incidence rates* per 1000 person-years were standardised for sex and age (categorised in 10-years groups). *HRs* adjusted (by multivariable Cox regression) for demographic characteristics (age, sex) and cumulated use of glucocorticoids. Reference: general population. *FU*, follow-up;*GenPop*, general population controls; *Std IR*, standardised incidence rate; TNFi, tumour necrosis factor inhibitor; yr, years.

The median follow-up time was: 5.0 years for the bionaïve patients with RA, 1.2 years for the TNFi-treated patients, 1.3 years for abatacept-treated patients, 2.2 years for rituximab-treated patients, 1.4 years for tocilizumab-treated patients and 4.4 years for the general population controls (figure 1). The longer median follow-up of bionaïve patients is explained by a majority entering the study in 2009 without being censored by starting a biological treatment. On the other hand, patients enter biologics cohorts evenly throughout the study period and exit more frequently due to treatment change.^40^

### Patients with RA vs general population

The sex-standardised and age-standardised incidence rate of lower GI perforation in the general population was 1.1 per 1000 person-years (95% CI 1.0 to 1.3). Compared to the general population, sex-standardised and age-standardised incidence rates of lower GI perforation were higher among patients with RA irrespective of cohort: 1.6 (95% CI 1.5 to 1.7) for bionaïve, 1.8 (1.4–3.6) for TNFi, 2.0 (1.3–5.7) for rituximab, 3.3 (1.7–16.6) for abatacept and 4.5 (2.7–10.4) for tocilizumab. Adjustment for cumulated GC use left only tocilizumab initiators at a higher risk than the general population (figure 1).

### Comparison of biological treatments

Before adjustment, the incidence rates of lower GI perforations were numerically higher for all three non-TNFi bDMARDs versus TNFi: abatacept 2.6 events per 1000 person-years (1.5–4.5), rituximab 2.1 (1.4–3.2), tocilizumab 4.1 (2.7–6.2) and TNFi 1.6 (1.2–2.1). After adjusting by IPTW for demographic characteristics (age, sex, education level), year of treatment start, disease history (GI perforations, diverticular disease, intestinal vascular disease, other GI disorders, inflammatory bowel disease, diabetes, chronic obstructive pulmonary disease, hospitalised infections, cardiovascular disease, cancer, joint surgery, number of hospitalisations), RA parameters (RA duration, rheumatoid factor, ESR, CRP, DAS28CRP, HAQ), comedication with methotrexate, other conventional DMARDs, selective COX2 inhibitors, NSAIDs, GCs and cumulated use of GCs and of NSAIDs, incidence rates for abatacept and rituximab dropped very close to TNFi, 2.0 (0.7–3.2) and 1.7 (0.8–2.5), respectively, versus 1.9 (1.3–2.4), leaving only tocilizumab with a more than double incidence rate of 4.1 (2.1–6.0) compared to TNFi (corresponding crude and adjusted HRs in table 2). In terms of numbers needed to harm, if 451 patients would be treated with tocilizumab instead of TNFi for 1 year, one extra GI perforation would be observed.

**Table 2:** Lower GI perforations, crude and IPTW-adjusted incidence rates and contrasts between non-TNFi and TNFi bDMARDs

Cohort	Crude IR (95% CI)	Crude HR (95% CI)	HR p value	IPTW adj. IR (95% CI)	IPTW adj. HR (95% CI)	HR p value
TNFi	1.57 (1.21–2.05)	Ref	‒	1.85 (1.34–2.36)	Ref	‒
Abatacept	2.62 (1.52–4.52)	1.68 (0.93–3.03)	0.0877	1.98 (0.73–3.23)	1.07 (0.55–2.10)	0.8341
Rituximab	2.11 (1.39–3.21)	1.36 (0.82–2.24)	0.2338	1.65 (0.84–2.46)	0.89 (0.50–1.58)	0.6980
Tocilizumab	4.10 (2.70–6.22)	2.61 (1.61–4.24)	0.0001	4.07 (2.14–6.00)	2.20 (1.28–3.79)	0.0045

IPTW adjustment for demographic characteristics (age, sex, education level), year of treatment start, disease history (GI perforations, diverticular disease, intestinal vascular disease, inflammatory bowel disease, other GI disorders, diabetes, chronic obstructive pulmonary disease, hospitalised infections, cardiovascular disease, cancer, joint surgery, number of hospitalisations), RA parameters (RA duration, rheumatoid factor, erythrocyte sedimentation rate CRP, DAS28CRP score), Health Assessment Questionnaire score, comedication with methotrexate, other conventional disease-modifying antirheumatic drugs, selective COX2 inhibitors, non-steroidal anti-inflammatory drugs (NSAIDs), glucocorticoids and cumulated use of glucocorticoids and of NSAIDs.

bDMARD, biological disease-modifying antirheumatic drugs; COX, cyclooxygenase; CRP, C reactive protein; GI, gastrointestinal; IPTW, inverse probability treatment weighting; IR, incidence rate; RA, rheumatoid arthritis; TNFi, tumour necrosis factor inhibitors.

After adjusting all confounders above by IPTW and line of therapy by conditioning on it, HRs dropped to 0.9 (0.4–1.9) for abatacept, 0.8 (0.4–1.6) for rituximab and 2.0 (1.1–3.5) for tocilizumab.

**Figure 2:**
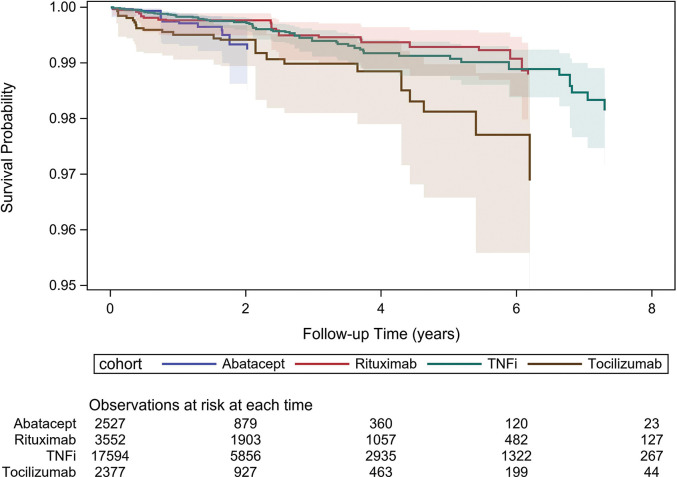
Adjusted lower gastrointenstinal perforations survival curves for biological disease-modifying antirheumatic drugs. Inverse probability of treatment weighting adjustment for demographic characteristics (age, sex, education level), year of treatment start, disease history (GI perforations, diverticular disease, intestinal vascular disease, inflammatory bowel disease, other GI disorders, diabetes, chronic obstructive pulmonary disease, hospitalised infections, cardiovascular disease, cancer, joint surgery, number of hospitalisations), RA parameters (RA duration, rheumatoid factor, erythrocyte sedimentation rate , CRP, Disease Activity Score using 28 joints and CRP score), Health Assessment Questionnaire score, comedication with methotrexate, other conventional DMARDs, selective COX2 inhibitors, non-steroidal anti-inflammatory drugs (NSAIDs), glucocorticoids and cumulated use of glucocorticoids and of NSAIDs. Lines represent point estimate survival functions while shaded areas represent 95% confidence limits around these estimates. COX, cyclooxygenase; CRP, C reactive protein; DMARD, disease-modifying antirheumatic drug; GI, gastro-intestinal; NSAID, non-steroidal anti-inflammatory drug; RA, rheumatoid arthritis.

The plot of adjusted survival estimates over time (figure 2) also shows a more accelerated decline in survival for tocilizumab compared to TNFi and rituximab, although with wide and overlapping confidence limits.

### Secondary analyses

Including both upper and lower GI perforations as outcome events, only slightly increased the number of events and incidence rates (online supplementary figure 2) compared to including only lower GI perforations. The contrasts between bDMARDs were also very similar to results obtained for lower GI perforations (online supplementary table 6).

Mimicking the specific definition validated by Curtis *et al*^41^ and used in two previous studies^14 17^ yielded too few events for stable incidence rates estimation or meaningful comparisons. Mimicking the broader definition used in the same studies yielded incidence rates and HRs comparable to our main results and to previous results (online supplementary table 7).

Out of 22 lower GI perforation cases identified for tocilizumab only 2 were fatal, corresponding to a 9.1% mortality. Mortality was lower among abatacept cases (7.7% (1/13)) and higher among rituximab (27.3% (6/22)) and TNFi (14% (8/57)). The difference in mortality between treatment cohorts was not statistically significant (p=0.36).

Excluding observations with any GI perforation before start of treatment left the results virtually unchanged. The crude incidence rate of lower GI perforation for abatacept was 2.5 (1.4–4.3), that of rituximab was 2.0 (1.3–3.1) and that of tocilizumab was 3.8 (2.4–5.8) compared to 1.5 (1.1–1.9) for TNFi.

## DISCUSSION

In this nationwide cohort study, to our knowledge the largest study so far assessing the incidence of GI perforations in different RA treatments in relation to the general population, we found an increased incidence of lower GI perforations in RA overall, seemingly explained by GC use. Meanwhile, we found a difference in risk between bDMARDs used to treat RA, corroborating earlier reports of an increased risk on tocilizumab, even when adjusted for a broad range of potential confounding factors.

As expected, the large majority of GI perforations (85%) were located in the lower GI tract in our material; thus, upper GI perforations were excluded from the main analyses. The sex-standardised and age-standardised incidence rates of lower GI perforations were higher among patients with RA compared to the general population, regardless of them being bionaïve or treated with bDMARDs. After adjusting for cumulated GC use before baseline, the risks in all RA cohorts, except tocilizumab initiators, were in line with the risk in the general population. Two previous studies compared the risk of GI perforation between patients with RA and age-matched and sex-matched patients who did not have RA. The first study included data from Olmsted County (US) residents and showed a doubled incidence rate of lower GI perforations among patients with RA.^1^ As the number of events was low, the CI of the rate ratio was wide and included the null, besides that no adjustment for GC exposure was made. Considering that tocilizumab was not yet approved during this study, a double risk for patients with RA is compatible with our results. In contrast to our results, the other study, using UK (Clinical Practice Research Datalink (CPRD) data, showed 35–60% higher incidence rates of GI perforation and bleeding among patients with RA, even after stratifying for having received GC prescriptions during follow-up.^2^ It is possible that the difference in outcome definitions explains the discrepancy, as we noted a tendency towards higher total (upper plus lower) GI perforation risk among bionaïve patients with RA (the largest RA cohort) compared to general population controls (online supplementary figure 2). Regardless, it seems likely that higher exposure to GCs among patients with RA is an important contributor to the overall increased risk of GI perforation.

Among patients with RA treated with bDMARDs, we observed a significantly increased risk of lower GI perforation among those who started tocilizumab compared with TNFi. This is in line with previous results,^14 16 17^ although there is variation in reported effect size. Whereas we report an adjusted HR of 2.2 (1.3–3.8) for lower GI perforations in tocilizumab versus TNFi, an adjusted HR of 4.5 (2.0–10.0) was observed in the German RABBIT registry when comparing tocilizumab versus csDMARDs,^16^ and two studies using US Health Insurance Claims data reported adjusted IRR of 4.0 (1.1–14.1)^14^ and HR of 2.5 (1.3–4.8)^17^ comparing tocilizumab versus TNFi. The differences between results may be within what is expected by chance, but there are also fundamental distinctions between studies. The German study validated outcome events initially reported by treating physicians, resulting in high specificity and lower incidence rates. While adverse events data collection and reporting by the treating rheumatologists might introduce surveillance bias, it is also true that severe adverse effects such as GI perforations are unlikely overlooked. Furthermore, the possibility of confounding adjustment was limited by potential underreporting of important comorbidities (such as diverticular disease) at baseline. The two US studies also used a narrower outcome definition that did not contain perforated diverticula. As approximately half the events observed in our data were perforated diverticula, our attempt to exclude them for comparison was uninformative. The US studies did present incidence rates of any GI perforation comparable to ours (and with highest rate on tocilizumab) from a broader definition that included perforated diverticula (online supplementary table 7). In addition to differences in which events were counted as GI perforations, studies used different data sources, coding systems, reference groups or exclusion criteria, making them unsuitable for direct meta-analysis. However, these differences could be seen as a strength since an increased risk of GI perforation for tocilizumab has now been observed in a range of settings.

The overall mortality in relation to a GI perforation event among bDMARD-treated patients with RA was 14.9% in our study, which was lower than previously reported.^16 17^ This may be explained by differences in study populations, case definitions or healthcare settings, but it may also reflect random variation. One previous study pointed to a potentially increased mortality after lower GI perforation among tocilizumab-treated patients,^16^ but this was not seen in the current study where the mortality among lower GI perforation cases treated with tocilizumab was not different compared to those treated with other bDMARDs.

Finally, we acknowledge a discrepancy between the current study and preliminary results presented by the authors as conference abstracts, where no statistically significant difference was found between treatment groups.^42^ The major driver of this difference was the addition of two years of data: 2009 and 2017.

Biological mechanisms that could explain the involvement of tocilizumab in GI perforation have been proposed. Amplified interleukin (IL)-6 expression has been observed early after GI injury, probably to support epithelial proliferation and wound healing.^43^ It has been reported that IL-6 regulates vascular endothelial growth factor production,^44^ which has a central role in angiogenesis and wound healing.^45^ By blocking IL-6 signalling, tocilizumab may slow down these healing processes. Additionally, tocilizumab treatment has also been associated with an increased incidence of diverticulitis.^46^ We also noticed a high proportion of diverticular perforations, out of identified lower GI perforations, among tocilizumab-treated patients (20/22).

Our study has several limitations. First, the outcomes were identified through national healthcare registries with overall high validity, but we are not aware of a validation specifically for GI perforations and false positives could have amplified incidence rates. Differential outcome detection could potentially bias our results if patients on treatments assumed to cause GI perforation (such as tocilizumab) are more closely monitored. On the other hand, detection bias could act in the opposite direction for tocilizumab, since IL-6 blockade may dampen perforation symptoms, making perforations difficult to detect. However, severe outcomes, such as GI perforations, are unlikely to be overlooked regardless of exposure history. Second, misclassification of bionaïve patients with RA is possible if they started treatments with a bDMARD not recorded in SRQ. Nonetheless, the great majority of bDMARD treatment starts are covered by SRQ and coverage increased over time. Third, there are important risk factors of GI perforation such as alcohol consumption, smoking or obesity that we had no data. While the pattern of alcohol consumption and smoking may differ between patients with RA and general population controls, it is not expected to be associated with the bDMARD choice. Furthermore, using an ‘on drug’ (‘as treated’) analysis is appropriate when we expect a limited latency time between exposure and the development of the outcome, but selection bias is possible if treatment is stopped due to prodromal symptoms of the outcome. To address this issue, we extended the exposure window over the end of treatment, assuming that it would not take more than 90 days for a GI perforation to be identified, once symptoms have been detected. Finally, the time-to-event analysis precludes an examination of patient experience after the first outcome event, such as stopping treatment due to GI perforation or reoccurrence of GI perforation. We chose a time-to-event analysis instead of a recurrent event analysis because it is difficult to recognise distinct GI perforation events from registry data. In order to address any possible bias due to events caused by a previous treatment being counted under a subsequent treatment, we conducted a sensitivity analysis including only patients without any history of GI perforation and allowing patients to re-enter follow-up only as long as they did not develop a lower GI perforation. The results were similar to the main analysis.

An important strength of our study is using national registers where data is recorded routinely, prospectively and independently of individual studies. This would counteract some differences in measurement error between compared exposures that would be present in unblinded, retrospective measurements. Because we could link the SRQ with other national Swedish registers, we had access to a wider range of patient characteristics than previous studies and to a sample of general population controls.

In conclusion, we found increased rates of lower GI perforations among patients with RA compared to the general population, seemingly explained by an increased use of GCs. We also found an increased rate of lower GI perforations on tocilizumab versus other bDMARDs. The absolute rates remained low, but considering the seriousness of GI perforations, even a slightly increased risk warrants caution when using tocilizumab.

**Key messages**

**What is already known about this subject?**

- Treatments for RA (including glucocorticoids), as well as the disease process itself, may be associated with an increased risk of gastrointestinal (GI) perforations.
- Limited data suggests an increased risk of lower GI perforations among patients treated with tocilizumab.

**What does this study add?**

- This cohort study used Swedish registers with national coverage to assess the risk of lower gastrointestinal perforations in RA per se and linked to biological therapies, adjusting for a broader range of confounding factors than previous studies.
- The rate of lower gastrointestinal perforations is increased among patients with RA compared to the general population, but this seems explained by the use of glucocorticoids rather than by the disease itself. An increased risk on tocilizumab compared with other biological treatments remained after adjustment.

**How might this impact on clinical practice?**

- The presence of other GI perforation risk factors should be assessed before tocilizumab administration and patients should be evaluated during treatment.

## References

[1] Myasoedova E, Matteson EL, Talley NJ, et al. Increased incidence and impact of upper and lower gastrointestinal events in patients with rheumatoid arthritis in Olmsted County, Minnesota: a longitudinal population-based study. *J Rheumatol* 2012;39:1355–62. 10.3899/jrheum.111311 22467929 PMC3389143

[2] Wilson JC, Sarsour K, Gale S, et al. Incidence and risk of glucocorticoid-associated adverse effects in patients with rheumatoid arthritis. *Arthritis Care Res (Hoboken)* 2019;71:498–511. 10.1002/acr.23611 29856128

[3] Hernández-Díaz S, Rodríguez LAG Steroids and risk of upper gastrointestinal complications. *Am J Epidemiol* 2001;153:1089–93. 10.1093/aje/153.11.1089 11390328

[4] Humes DJ, Fleming KM, Spiller RC, et al. Concurrent drug use and the risk of perforated colonic diverticular disease: a population-based case: control study. *Gut* 2011;60:219–24. 10.1136/gut.2010.217281 20940283

[5] Sostres C, Gargallo CJ, Lanas A Nonsteroidal anti-inflammatory drugs and upper and lower gastrointestinal mucosal damage. *Arthritis Res Ther* 2013;15:S3 10.1186/ar4175. PMC3890944 24267289

[6] Curtis JR, Xie F, Chen L, et al. The incidence of gastrointestinal perforations among rheumatoid arthritis patients. *Arthritis Rheum* 2011;63:346–51. 10.1002/art.30107 20967860 PMC3031757

[7] Corsi F, Previde P, Colombo F, et al. Two cases of intestinal perforation in patients on anti-rheumatic treatment with etanercept. *Clin Exp Rheumatol* 2006;24:113. 16539834

[8] European Medicines Agency Remicade. European Medicines Agency 2018 Available https://www.ema.europa.eu/en/documents/product-information/remicade-epar-product-information_en.pdf (accessed 20 Jan 2020)

[9] European Medicines Agency Humira. European Medicines Agency 2020 Available https://www.ema.europa.eu/en/documents/product-information/humira-epar-product-information_en.pdf (accessed 20 Jan 2020).

[10] European Medicines Agency Cimzia. European Medicines Agency 2019 Available https://www.ema.europa.eu/en/documents/product-information/cimzia-epar-product-information_en.pdf (accessed 20 Jan 2020)

[11] Závada J, Lunt M, Davies R, et al. The risk of gastrointestinal perforations in patients with rheumatoid arthritis treated with anti-TNF therapy: results from the BSRBR-RA. *Ann Rheum Dis* 2014;73:252–5. 10.1136/annrheumdis-2012-203102 23644671

[12] Jagpal A, Curtis JR Gastrointestinal perforations with biologics in patients with rheumatoid arthritis: implications for clinicians. *Drug Saf* 2018;41:545–53 10.1007/s40264-018-0639-1. 29392593

[13] Schiff MH, Kremer JM, Jahreis A, et al. Integrated safety in tocilizumab clinical trials. *Arthritis Res Ther* 2011;13:R141 10.1186/ar3455 PMC3308069 21884601

[14] Monemi S, Berber E, Sarsour K, et al. Incidence of gastrointestinal perforations in patients with rheumatoid arthritis treated with tocilizumab from clinical trial, postmarketing, and real-world data sources. *Rheumatol Ther* 2016;3:337–52. 10.1007/s40744-016-0037-z 27747579 PMC5127961

[15] Curtis JR, Perez-Gutthann S, Suissa S, et al. Tocilizumab in rheumatoid arthritis: a case study of safety evaluations of a large postmarketing data set from multiple data sources. *Semin Arthritis Rheum* 2015;44:381–8. 10.1016/j.semarthrit.2014.07.006 25300699

[16] Strangfeld A, Richter A, Siegmund B, et al. Risk for lower intestinal perforations in patients with rheumatoid arthritis treated with tocilizumab in comparison to treatment with other biologic or conventional synthetic DMARDs. *Ann Rheum Dis* 2017;76:504–10. 10.1136/annrheumdis-2016-209773 27405509 PMC5445993

[17] Xie F, Yun H, Bernatsky S, et al. Brief report: risk of gastrointestinal perforation among rheumatoid arthritis patients receiving tofacitinib, tocilizumab, or other biologic treatments. *Arthritis Rheumatol (Hoboken, NJ)* 2016;68:2612–7. 10.1002/art.39761 PMC5538140 27213279

[18] Frisell T, Baecklund E, Bengtsson K, et al. Patient characteristics influence the choice of biological drug in RA, and will make non-TNFi biologics appear more harmful than TNFi biologics. *Ann Rheum Dis* 2018;77:650–7. 10.1136/annrheumdis-2017-212395 29237621 PMC5909744

[19] Ludvigsson JF, Andersson E, Ekbom A, et al. External review and validation of the Swedish national inpatient register. *BMC Public Health* 2011;11:450 10.1186/1471-2458-11-450 PMC3142234 21658213

[20] Eriksson JK, Askling J, Arkema EV The Swedish rheumatology quality register: optimisation of rheumatic disease assessments using register-enriched data. *Clin Exp Rheumatol* 2014;32:S–147–149. 25365105

[21] Wadström H, Eriksson JK, Neovius M, et al. How good is the coverage and how accurate are exposure data in the Swedish biologics register (ARTIS)? *Scand J Rheumatol* 2015;44:22–8. 10.3109/03009742.2014.927918 25379815

[22] Wettermark B, Hammar N, MichaelFored C, et al. The new Swedish prescribed drug register: opportunities for pharmacoepidemiological research and experience from the first six months. *Pharmacoepidemiol Drug Saf* 2007;16:726–35. 10.1002/pds.1294 16897791

[23] Ludvigsson JF, Almqvist C, Bonamy A-KE, et al. Registers of the Swedish total population and their use in medical research. *Eur J Epidemiol* 2016;31:125–36. 10.1007/s10654-016-0117-y 26769609

[24] Brooke HL, Talbäck M, Hörnblad J, et al. The Swedish cause of death register. *Eur J Epidemiol* 2017;32:765–73. 10.1007/s10654-017-0316-1 28983736 PMC5662659

[25] Ludvigsson JF, Otterblad-Olausson P, Pettersson BU, et al. The Swedish personal identity number: possibilities and pitfalls in healthcare and medical research. *Eur J Epidemiol* 2009;24:659–67. 10.1007/s10654-009-9350-y 19504049 PMC2773709

[26] Waldenlind K, Eriksson JK, Grewin B, et al. Validation of the rheumatoid arthritis diagnosis in the Swedish national patient register: a cohort study from Stockholm county. *BMC Musculoskelet Disord* 2014;15:432 10.1186/1471-2474-15-432 PMC4302140 25510838

[27] Berglund P and Heeringa SG General theory for multiple imputation algorithms In: *Multiple imputation of missing data using SAS*. SAS Institute, 2014.

[28] White IR, Royston P Imputing missing covariate values for the Cox model. *Stat Med* 2009;28:1982–98 10.1002/sim.3618. 19452569 PMC2998703

[29] Morisot A, Bessaoud F, Landais P, et al. Prostate cancer: net survival and cause-specific survival rates after multiple imputation. *BMC Med Res Methodol* 2015;15:54 10.1186/s12874-015-0048-4 PMC4517373 26216355

[30] Fay MP, Feuer EJ Confidence intervals for directly standardized rates: a method based on the gamma distribution. *Stat Med* 1997;16:791–801. 9131766 10.1002/(sici)1097-0258(19970415)16:7<791::aid-sim500>3.0.co;2-#

[31] Austin PC, Stuart EA Moving towards best practice when using inverse probability of treatment weighting (IPTW) using the propensity score to estimate causal treatment effects in observational studies. *Stat Med* 2015;34:3661–79 10.1002/sim.6607. 26238958 PMC4626409

[32] McCaffrey DF, Griffin BA, Almirall D, et al. A tutorial on propensity score estimation for multiple treatments using generalized boosted models. *Stat Med* 2013;32:3388–414. 10.1002/sim.5753 23508673 PMC3710547

[33] Cole SR, Hernán MA Adjusted survival curves with inverse probability weights. *Comput Methods Programs Biomed* 2004;75:45–9. 10.1016/j.cmpb.2003.10.004 15158046

[34] Joffe MM, Have TRT, Feldman HI, et al. Model selection, confounder control, and marginal structural models. *Am Stat* 2004;58:272–9. 10.1198/000313004X5824

[35] Lin DY, Wei LJ The robust inference for the cox proportional hazards model. *J Am Stat Assoc* 1989;84:1074–8 10.1080/01621459.1989.10478874.

[36] Austin PC Variance estimation when using inverse probability of treatment weighting (IPTW) with survival analysis: variance estimation for IPTW with survival outcomes. *Stat Med* 2016;35:5642–55 10.1002/sim.7084. 27549016 PMC5157758

[37] WHOCC - Definition and general considerations Available https://www.whocc.no/ddd/definition_and_general_considera/ (accessed 30 Mar 2020)

[38] Cole SR, Hernán MA Constructing inverse probability weights for marginal structural models. *Am J Epidemiol* 2008;168:656–64. 10.1093/aje/kwn164 18682488 PMC2732954

[39] Hernán MA, Robins JM Effect modification and marginal structural models In: *Causal inference: what if*. Boca Raton, FL: Chapman & Hall/CRC, 2020: 157–8.

[40] Frisell T, Dehlin M, Di Giuseppe D, et al. Comparative effectiveness of abatacept, rituximab, tocilizumab and TNFi biologics in RA: results from the nationwide Swedish register. *Rheumatology (Oxford)* 2019;58:1367–77. 10.1093/rheumatology/key433 30668875

[41] Curtis JR, Chen S-Y, Werther W, et al. Validation of ICD-9-CM codes to identify gastrointestinal perforation events in administrative claims data among hospitalized rheumatoid arthritis patients. *Pharmacoepidemiol Drug Saf* 2011;20:1150–8. 10.1002/pds.2215 22020901 PMC3227025

[42] Barbulescu A *The risk of gastrointestinal perforations associated with biologic disease-modifying anti-rheumatic drugs used in rheumatoid arthritis: a nationwide Swedish cohort study*. ACR, 2018 Available https://acr.confex.com/acr/2018/meetingapp.cgi/Paper/73624 (accessed 26 Mar 2020)

[43] Kuhn KA, Manieri NA, Liu T-C, et al. IL-6 stimulates intestinal epithelial proliferation and repair after injury. *PLoS One* 2014;9:e114195 10.1371/journal.pone.0114195 PMC4257684 25478789

[44] Nakahara H, Song J, Sugimoto M, et al. Anti-interleukin-6 receptor antibody therapy reduces vascular endothelial growth factor production in rheumatoid arthritis. *Arthritis Rheum* 2003;48:1521–9. 10.1002/art.11143 12794819

[45] Verheul HMW, Pinedo HM Possible molecular mechanisms involved in the toxicity of angiogenesis inhibition. *Nat Rev Cancer* 2007;7:475–85. 10.1038/nrc2152 17522716

[46] Pawar A, Desai RJ, Solomon DH, et al. Risk of serious infections in tocilizumab versus other biologic drugs in patients with rheumatoid arthritis: a multidatabase cohort study. *Ann Rheum Dis* 2019;78:456–64. 10.1136/annrheumdis-2018-214367 30679153

