# Supplementary material for: Gastrointestinal perforations in patients with rheumatoid arthritis treated with biological disease-modifying antirheumatic drugs in Sweden: a nationwide cohort study
Source: RMD Open. 2020 Jul 14;6(2):e001201. doi: 10.1136/rmdopen-2020-001201 (PMC7425111; doi:10.1136/rmdopen-2020-001201)
Supplement: Supplementary data [file rmdopen-2020-001201s001.pdf]

Supplementary material for article:

Gastro-intestinal perforations in rheumatoid arthritis patients treated with biological disease-modifying anti-rheumatic drugs in Sweden: a nationwide cohort study

Andrei Barbulescu[1], MMSc ; Bénédicte Delcoigne[1], PhD; Johan Askling[1,2], MD/PhD; Thomas Frisell[1], PhD on behalf of the ARTIS Study group

[1]Clinical Epidemiology Division, Department of Medicine, Solna, Karolinska Institutet, Stockholm, Sweden
[2]Department of Rheumatology, Karolinska University Hospital, Stockholm, Sweden

Contents:

Supplementary Table 1 – Outcome definitions

Supplementary Table 2 – Number and location of gastro-intestinal (GI) perforation events

Supplementary Table 3 – Missing data in the sample of bDMARD treated patients and distribution of each variable with missing values before and after imputation

Supplementary Table 4 – Definitions of baseline covariates

Supplementary Table 5 – Distribution of characteristics in the bDMARD treated sample, before / after weighting

Supplementary Table 6 – Any (lower or upper) GI perforation crude and IPTW adjusted incidence rates and contrasts between non-TNFi and TNFi bDMARDs

Supplementary Table 7 – Crude incidence rates of any (lower or upper) GI perforation, obtained in different settings using the sensitive definition employed by Xie et al.[1] and applying similar exclusion criteria

Supplementary Figure 1 – Distribution of *Inverse Probability of Treatment Weights* (IPTW) within each imputed data-set and overall

Supplementary Figure 2 – Any (lower or upper) GI perforation sex- age- standardized incidence rates and adjusted contrasts between RA patients and the general population

Supplementary Table 1 – Outcome definitions

Location	Description	ICD10 codes	Procedure codes [a]	Definitions [c]
Upper GI	Perforation of oesophagus	K223		A, X1, X2
Upper GI	Gastric ulcer with perforation	K251 K252 K255 K256		A, X1, X2
Upper GI	Duodenal ulcer with perforation	K261 K262 K265 K266		A, X1, X2
Upper GI	Peptic ulcer with perforation	K271 K272 K275 K276		A, X1, X2
Upper GI	Fistula of stomach or duodenum	K316		A
Lower GI	Gastro-jejunal ulcer with perforation	K281 K282 K285 K286		A, X1, X2
Lower GI	Diverticular disease with perforation	K570 K572 K574 K578		A, X2
Lower GI	Diverticular disease without perforation – small intestine	K571	JFB01 JFB21 JFC01 JFC11 JFA74 JFF01 JFF11 JFF24 JFF21 JFC21 JFC41 JFA00 JFA73 JFA76 JFB00 JFB20 JFC00 JFC10 JFF00 JFF10 JFF13 JFF23 JFF20 JFC20 JFC40 JFA70 JFA71 JFA76	X2
Lower GI	Diverticular disease without perforation – large intestine	K573	JFA84 JFB31 JFB41 JFB44 JFB47 JFB51 JFB61 JFB64 JFC51 JFF41 JFF51 JFC31 JFC51 JFF24 JFF27 JFF31 JFF41 JFF51 JFA83 JFB30 JFB40 JFB43 JFB46 JFB50 JFB60 JFB63 JFC30 JFC50 JFF23 JFF26 JFF30 JFF40 JFF50 JFF60 JFA80 JFA81 JFA86	X2
Lower GI	Diverticular disease without perforation – both small and large intestine or part unspecified	K575 K579	JFB01 JFB21 JFC01 JFC11 JFA74 JFF01 JFF11 JFF24 JFF21 JFC21 JFC41 JFA00 JFA73 JFA76 JFB00 JFB20 JFC00 JFC10 JFF00 JFF10 JFF13 JFF23 JFF20 JFC20 JFC40 JFA70 JFA71 JFA76 JFA84 JFB31 JFB41 JFB44 JFB47 JFB51 JFB61 JFB64 JFC51 JFF41 JFF51 JFC31 JFC51 JFF24 JFF27 JFF31 JFF41 JFF51 JFA83 JFB30 JFB40 JFB43 JFB46 JFB50 JFB60 JFB63 JFC30 JFC50 JFF23 JFF26 JFF30 JFF40 JFF50 JFF60 JFA80 JFA81 JFA86	X2
Lower GI	Perforation of intestine	K631		A, X1, X2
Lower GI	Fistula of intestine	K632		A
Lower GI	Abscess of intestine	K630		A
Lower GI	Appendicitis with perforation/rupture and generalized peritonitis	K352 K350 [b]		A, X2
Lower GI	Appendicitis with or without perforation/rupture and localized peritonitis	K353 K351 [b]		A
Lower GI	Vascular disorder of intestine	K550 K551 K559	JFB01 JFB21 JFC01 JFC11 JFA74 JFF01 JFF11 JFF24 JFF21 JFC21 JFC41 JFA00 JFA73 JFA76 JFB00 JFB20	X2

Supplementary Table 1 – Outcome definitions

Location	Description	ICD10 codes	Procedure codes [a]	Definitions [c]
			JFC00 JFC10 JFF00 JFF10 JFF13 JFF23 JFF20 JFC20 JFC40 JFA70 JFA71 JFA76 JFA84 JFB31 JFB41 JFB44 JFB47 JFB51 JFB61 JFB64 JFC51 JFF41 JFF51 JFC31 JFC51 JFF24 JFF27 JFF31 JFF41 JFF51 JFA83 JFB30 JFB40 JFB43 JFB46 JFB50 JFB60 JFB63 JFC30 JFC50 JFF23 JFF26 JFF30 JFF40 JFF50 JFF60 JFA80 JFA81 JFA86	

[a] The Swedish version of NOMESCO – Classification of Surgical Procedures, version 1.9 is used.

[b] Old versions of ICD-10 codes used until 2010.

[c] “A” refers to the definition used in the current study; “X1” refers to the emulation of the specific definition employed by Xie et al.[1]; “X2” refers to the emulation the sensitive definition employed by Xie et al.[1]

Supplementary Table 2 – Number and location of gastro-intestinal (GI) perforation events

		Location of GI perforation		
**Cohort**	**Number of GI perforations**	**Lower GI (%)**	**Upper GI (%)**	**Both upper and lower GI (%)**
Overall	*1186*	*84.7*	*15.1*	*0.2*
General Population	374	88.5	11.5	0
Bionaïve	692	80.9	18.8	0.3
Abatacept	15	86.7	13.3	0
Rituximab	23	95.7	4.3	0
TNFi	59	96.6	3.4	0
Tocilizumab	23	91.3	4.3	4.4

Supplementary Table 3 –Missing data in the sample of bDMARD treated patients and distribution of each variable with missing values before and after imputation

**Variable**	**Missingness** N (%)	**Distribution before imputation** (% for binary variables, mean (min-max) for continuous variables)	**Distribution after imputation** (% for binary variables, mean (min-max) for continuous variables)
Highest Education (9 years or less)	230 (0.88)	23.3	23.4
Highest Education (10 to 12 years)	230 (0.88)	47.6	47.6
Highest Education (more than 12 years)	230 (0.88)	29.1	29.0
Rheumatoid Factor	707 (2.71)	78.1	78.1
RA duration	246 (0.94)	12.7 (0.0 - 97.2)	12.7 (0.0 - 97.2)
ESR	7443 (28.57)	26.81 (1.00 - 488.00)	25.20 (1.00 - 488.00)
CRP	6130 (23.53)	16.48 (0.00 - 286.00)	15.98 (0.00 - 286.00)
DAS28CRP score	8209 (31.51)	4.41 (1.03 - 8.72)	4.34 (1.03 - 8.72)
HAQ score	8113 (31.14)	1.12 (0.00 - 3.00)	1.10 (0.00 - 3.00)
MTX co-medication	4860 (18.66)	62.0	61.9
Other DMARD co-medication	4860 (18.66)	13.0	12.9
COX2 inhibitors co-medication	4860 (18.66)	2.6	2.6
NSAIDs co-meditation	4860 (18.66)	32.1	31.4
Glucocorticoid co-medication	4860 (18.66)	49.8	48.4

The distribution of variables is represented as percentage for binary variables and as mean (minimum – maximum) for continuous variables.

Abbreviations: COX = cyclooxygenase; CRP = C-reactive protein; DAS28CRP = Disease Activity Score using 28 joints and CRP; DMARD= disease-modifying anti-rheumatic drug; ESR = erythrocyte sedimentation rate; HAQ = health assessment questionnaire; NSAID = non-steroidal anti-inflammatory drug

Supplementary Table 4 – Definitions of baseline covariates

Characteristic	Period of measurement	Source	Definition/Transformation
Demographic characteristics: age, sex, death date	At baseline	Total population register	The *age* at baseline was calculated using the recorded *birthdate* and the *start of treatment*.
Highest level of education achieved at baseline	At baseline	Education Register	Categorized into:<br>(1) Less than 9 years<br>(2) 10 to 12 years<br>(3) More than 12 years
RA characteristics:<br>Rheumatoid factor (RF);<br>ESR; CRP; DAS28CRP;<br>HAQ	60 days before and 20 days after baseline. Pick the closest visit to baseline.	Swedish Rheumatology Quality register (SRQ)	RF is binary (positive/negative); ESR, CRP, DAS28CRP are continuous; HAQ is discrete (treated as continuous).
Co-medication:<br>Methotrexate;<br>Other csDMARD;<br>COX2 inhibitor;<br>Non-selective NSAID;<br>Glucocorticoid	60 days before and 20 days after baseline. Pick the closest visit to baseline.	Swedish Rheumatology Quality register (SRQ)	All quantities are binary (present/absent). If a prescription was recorded for the visit the value is *present* (1). Otherwise the value is *absent* (0).
Cumulated exposure to glucocorticoids and NSAIDs	1 year before baseline	Prescribed drugs register	Total dispensed amount[1]. Categorized.
GI perforation	5 years before baseline	National Patient Register (inpatient and outpatient)	Binary (yes/no).<br>ICD10 codes:<br>K223 K251 K252 K255 K256 K261 K262 K265 K266 K271 K272 K275 K276 K316 K281 K282 K285 K286 K570 K572 K574 K578 K630 K631 K632 K352 K350 K351
Diverticular disease	5 years before baseline	National Patient Register (inpatient and outpatient)	Binary (yes/no).<br>ICD10 codes:<br>K57 Q430
GI ischemia	5 years before baseline	National Patient Register (inpatient and outpatient)	Binary (yes/no).<br>ICD10 codes:<br>K550 K551 K559
Other GI conditions	5 years before baseline	National Patient Register (inpatient and outpatient)	Binary (yes/no).<br>ICD10 codes:<br>K20 K21 K221 K227 K250 K253 K254 K257 K259 K260 K263 K264 K267 K269 K270 K273 K274 K277 K279 K280 K283 K284 K287 K289 K29 K314 K315 K52 K56 K58 K633 K590 K593
IBD (Inflammatory bowel disease)	5 years before baseline	National Patient Register (inpatient and outpatient)	Binary (yes/no).<br>ICD10 codes:<br>K50 K51
Diabetes	5 years before baseline	National Patient Register (inpatient and outpatient)	Binary (yes/no).<br>ICD10 codes:<br>E10 E11 E12 E13 E14 O24
COPD (Chronic Obstructive Pulmonary Disease)	5 years before baseline	National Patient Register (inpatient and outpatient)	Binary (yes/no).<br>ICD10 codes:<br>J41 J42 J43 J44
CVD (Cardiovascular Vascular Disease)	5 years before baseline	National Patient Register (inpatient and outpatient)	Binary (yes/no).<br>ICD10 codes:

Supplementary Table 4 – Definitions of baseline covariates

Characteristic	Period of measurement	Source	Definition/Transformation
			I1 I2 I3 I4 I5 I6
Cancer	Ever before baseline	Cancer registry	
Hospitalized Infections	1 year before baseline	National Patient Register (inpatient)	Binary (yes/no).<br>ICD10 codes:<br>A B G00 G01 G02 G042 G05 G06 G07 H600 H603 H620 H621 H622 H623 H66 H67 H70 H940 J0 J1 J2 J32 J340 J36 J383 J390 J391 K102 L0 M00 M01 M462 M463 M464 M465 M86 N10 N410 N412 N431 N450 N74 N771 N300 O98
Surgical interventions on joints	5 years before baseline	National Patient Register (inpatient, outpatient)	Binary (yes/no).<br>Procedure codes:<br>NGB, 8423-8424, 8426<br>NFB, 8400-8415, 8419<br>NBB, 8437<br>NHB, NHC, NHE, NHF, NHG, 8420-8422, 8436<br>NDB, NDC, NDE, NDF, NDG
Number of hospitalizations	5 years before baseline	National Patient Register (inpatient)	Categorized

[1] To be able to cumulate dispensed quantities of different glucocorticoids or NSAIDs, these were transformed into numbers of “defined daily doses” (DDDs). One DDD represents the quantity of drug used per day by an average patient for the main indication of the drug.[2]

Supplementary Table 5 – Distribution of characteristics in the bDMARD treated sample, before / after weighting.

Feature	All	TNFi	Abatacept	Rituximab	Tocilizumab
Female	76.50 / 76.52	75.71 / 76.53	80.37 / 76.93	75.79 / 76.52	79.30 / 76.01
Age	57.87 / 57.92	56.82 / 57.91	59.46 / 57.7	62.17 / 58.16	57.49 / 57.88
Edu. (≤ 9 years)	23.40 / 23.59	22.44 / 23.45	24.32 / 23.93	27.64 / 23.77	23.12 / 23.96
Edu. (10-12 years)	47.59 / 47.63	47.45 / 47.62	49.12 / 47.61	46.85 / 47.28	48.06 / 48.26
Edu (> 12 years)	29.02 / 28.78	30.11 / 28.92	26.56 / 28.47	25.50 / 28.95	28.82 / 27.78
GI perforation	0.59 / 0.59	0.45 / 0.58	1.15 / 0.58	0.87 / 0.59	0.59 / 0.64
Diverticular Dis.	3.25 / 3.26	2.73 / 3.27	4.79 / 3.20	4.53 / 3.14	3.53 / 3.41
Intestinal Vasc. Dis.	0.07 / 0.06	0.06 / 0.06	0.08 / 0.05	0.11 / 0.05	0.04 / 0.09
Other GI Dis.	9.62 / 9.81	8.49 / 9.82	12.86 / 9.79	12.56 / 9.56	10.18 / 10.12
IBD	1.32 / 1.37	1.34 / 1.34	1.46 / 1.23	1.13 / 1.40	1.35 / 1.64
Diabetes	7.52 / 7.69	6.71 / 7.66	9.73 / 7.76	9.88 / 8.00	7.66 / 7.38
COPD	3.07 / 3.09	2.36 / 3.07	5.07 / 3.13	5.07 / 3.17	3.24 / 3.07
Hospitalized Infection	4.42 / 4.52	2.96 / 4.48	8.55 / 4.49	8.39 / 4.67	4.96 / 4.66
Cardiovascular Dis.	25.52 / 25.89	22.38 / 25.72	32.45 / 25.51	35.11 / 27.25	27.05 / 25.56
Cancer	6.79 / 6.98	5.25 / 6.96	7.32 / 6.82	14.70 / 7.26	5.85 / 6.88
Joint Surgery	16.81 / 16.99	14.37 / 16.92	21.13 / 16.68	23.23 / 17.49	20.66 / 17.14
N hospitalizations	1.54 / 1.56	1.24 / 1.53	2.20 / 1.60	2.37 / 1.65	1.86 / 1.60
RA duration	12.69 / 12.69	11.88 / 12.7	14.41 / 12.53	15.16 / 12.82	13.20 / 12.36
Rheumatoid Factor	78.08 / 78.04	75.93 / 78.09	79.20 / 77.75	87.50 / 78.31	78.77 / 77.65
ESR	25.19 / 25.52	22.85 / 25.35	26.60 / 25.97	31.51 / 26.05	31.59 / 25.4
CRP	15.97 / 16.2	14.20 / 16.08	16.41 / 16.83	20.31 / 16.80	22.15 / 16.31
DAS28CRP score	4.34 / 4.36	4.19 / 4.35	4.58 / 4.37	4.64 / 4.37	4.80 / 4.38
HAQ score	1.10 / 1.11	1.02 / 1.12	1.29 / 1.11	1.27 / 1.12	1.28 / 1.12
Methotrexate	61.91 / 61.65	65.50 / 61.71	55.76 / 60.90	56.22 / 61.65	50.36 / 62.00
Other csDMARDs	12.88 / 12.82	13.14 / 12.83	10.36 / 13.38	15.28 / 12.49	10.01 / 12.57
COX2 inhibitors	2.63 / 2.60	2.64 / 2.61	2.63 / 2.51	2.31 / 2.47	3.00 / 2.76
NSAID baseline	31.39 / 31.26	30.57 / 31.26	32.58 / 31.14	31.65 / 31.34	35.85 / 31.28
GC baseline	48.43 / 48.72	44.62 / 48.54	56.62 / 48.81	57.38 / 49.52	54.52 / 48.79
Cum. NSAID	159.0 / 159.1	158. / 159.1	156.5 / 160.3	152.6 / 159.2	171.8 / 158.1
Cum. GC	125.3 / 126.1	112.5 / 124.4	148.5 / 128.1	157.2 / 131.0	148.2 / 129.1
Start 2009	8.68 / 8.61	8.68 / 8.61	4.99 / 8.24	12.44 / 8.94	6.94 / 8.52
Start 2010	9.83 / 9.85	9.77 / 9.90	6.96 / 9.40	11.77 / 10.03	10.48 / 9.74
Start 2011	10.06 / 10.10	10.05 / 10.07	7.95 / 10.28	11.35 / 10.27	10.43 / 9.91
Start 2012	9.53 / 9.58	9.17 / 9.57	8.43 / 8.89	11.20 / 9.66	10.81 / 10.25
Start 2013	10.37 / 10.44	9.69 / 10.40	14.48 / 11.01	10.44 / 10.48	10.90 / 10.04
Start 2014	10.50 / 10.60	9.88 / 10.55	14.17 / 10.96	10.11 / 10.63	11.82 / 10.57
Start 2015	11.05 / 11.01	10.29 / 10.99	14.60 / 11.31	11.09 / 10.67	12.83 / 11.36
Start 2016	15.17 / 15.11	16.34 / 15.19	14.44 / 15.26	11.26 / 14.46	13.13 / 15.25
Start 2017	14.81 / 14.71	16.12 / 14.73	13.97 / 14.65	10.33 / 14.87	12.66 / 14.36

Distribution parameters: mean for continuous variables and proportion for binary variables.

Analysis is performed within each imputation, thus IPTWs are estimated within each imputation and balance is also achieved within each imputation. However, to ease presentation, means/proportions pooled over all imputations are presented in the table.

The “All” column represents distributions in the entire sample (marginal). Marginal distributions are not changed by weighting.

The following variables were included in the inverse probability of treatment weight denominator (thus adjusted): demographic characteristics (age, sex, education level), year of treatment start, disease history (GI perforations, diverticular disease, intestinal vascular disease, inflammatory bowel disease, other GI disorders, diabetes, chronic obstructive pulmonary disease, hospitalized infections, cardio-vascular disease, cancer, joint surgery, number of hospitalizations) RA parameters (RA duration, rheumatoid factor, ESR, CRP, DAS28CRP score), health assessment questionnaire score, co-mediation with: methotrexate, other conventional DMARDs, selective COX2 inhibitors, NSAIDs, glucocorticoids and cumulated use of glucocorticoids and of NSAIDs.

Abbreviations: COPD = chronic obstructive pulmonary disease; COX = cyclooxygenase; CRP = C-reactive protein; csDAS28CRP = conventional synthetic Disease Activity Score using 28 joints and CRP; DMARD= disease-modifying anti-rheumatic drug; ESR = erythrocyte sedimentation rate; GI = gastro-intestinal; HAQ = health assessment questionnaire; IBD = inflammatory bowel disease; NSAID = non-steroidal anti-inflammatory drug

Supplementary Table 6 – Any (lower or upper) GI perforation, crude and IPTW adjusted incidence rates and contrasts between non-TNFi and TNFi bDMARDs

Cohort	Crude IR (95% CI)	Crude HR (95% CI)	HR p-value	IPTW adj. IR (95% CI)	IPTW adj. HR (95% CI)	HR p-value
TNFi	1.63 (1.26-2.11)	Ref	-	1.99 (1.43-2.56)	Ref	-
Abatacept	3.03 (1.82-5.03)	1.87 (1.07-3.27)	0.0280	2.19 (0.84-3.53)	1.10 (0.57-2.12)	0.7829
Rituximab	2.21 (1.47-3.32)	1.36 (0.84-2.22)	0.2144	1.80 (0.91-2.69)	0.90 (0.51-1.60)	0.7237
Tocilizumab	4.28 (2.85-6.45)	2.64 (1.64-4.24)	0.0001	4.21 (2.25-6.16)	2.11 (1.23-3.61)	0.0066

IPTW adjustment for: demographic characteristics (age, sex, education level), year of treatment start, disease history (GI perforations, diverticular disease, intestinal vascular disease, inflammatory bowel disease, other GI disorders, diabetes, chronic obstructive pulmonary disease, hospitalized infections, cardio-vascular disease, cancer, joint surgery, number of hospitalizations) RA parameters (RA duration, rheumatoid factor, ESR, CRP, DAS28CRP, health assessment questionnaire), co-medication with: methotrexate, other conventional DMARDs, selective COX2 inhibitors, NSAIDs, glucocorticoids and cumulated use of glucocorticoids and of NSAIDs.

Abbreviations: bDMARD=biological disease-modifying anti-rheumatic drugs, CI=confidence interval, HR=hazard ratios, IPTW=inverse probability treatment weighting, IR=incidence rate, TNFi = tumour-necrosis-factor inhibitors.

Supplementary Table 7 – Crude incidence rates of any (lower or upper) GI perforation, obtained in different settings using the sensitive definition employed by Xie et al.[1] and applying similar exclusion criteria

	**Definition emulated in current data**			**Xie et al.**[1]	**Monemi et al.**[3]
**Cohort**	**Person years**	**No. events**	**IR [a] (95% CI)**	**IR [a] (95% CI)**	**IR [a] (95% CI)**
TNFi	33,989.19	40	1.18 (0.86-1.60)	1.64 (1.43–1.87)	0.9 (0.5–1.5)
Abatacept	4534.84	9	1.98 (1.03-3.82)	1.81 (1.44–2.29)	1.4 (0.6–2.7)
Rituximab	8805.17	16	1.82 (1.11-2.97)	1.94 (0.97–3.87)	-
Tocilizumab	4951.80	19	3.84 (2.45-6.01)	2.53 (1.72–3.71)	2.8 (1.3–5.2)

[a] IR = crude incidence rates measured per 1000 person-years.

Xie et al.[1] analysed data of RA patients from Medicare (2006–2013) and MarketScan (2010–2014). Monemi et al.[3] analysed data of RA patients from MarketScan (2010-2014). Both studies employed GI perforations definitions, previously published by Curtis et al.[4]. Both studies excluded patients with a history of GI perforation, IBD or cancer. We attempted to emulate the outcome definitions used in these studies, translating ICD9CM to ICD10 and CPT to NOMESCO, and using our data (see definitions X1 and X2 in **Supplementary Table 1**). The *specific (narrow) definition* (X1) yielded too few events for stable estimates. Above we compare results obtained using the *sensitive (broader) definition* (X2).

Supplementary Figure 1 – Distribution of *Inverse Probability of Treatment Weights* (IPTW) within each imputed data-set and overall

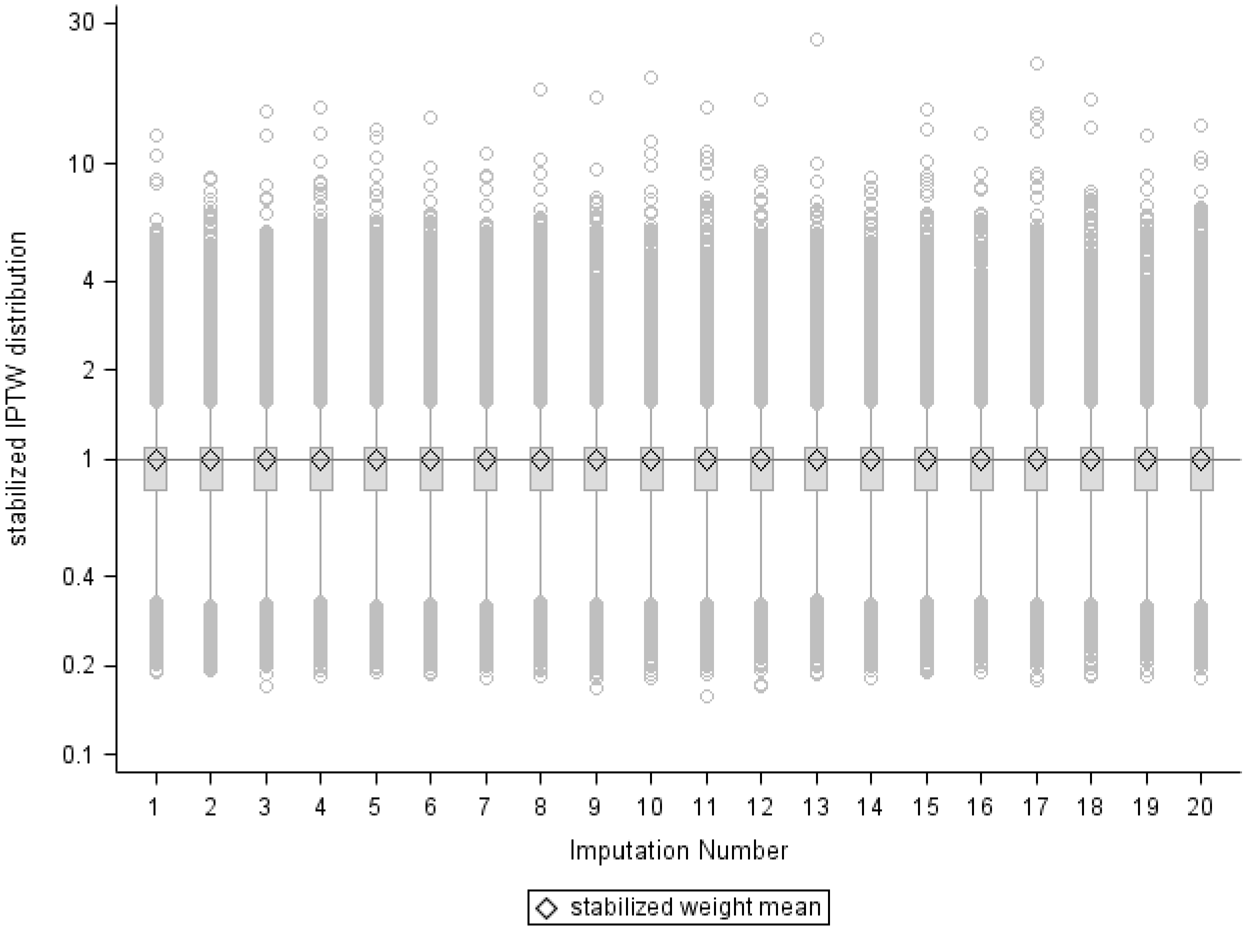

The box plots represent the distribution of IPTW within each imputed data-set

Overall distribution of IPTW

Minimum	Q1 (25%)	Median	Mean	Q3 (75%)	Max
0.16	0.79	0.90	0.99	1.10	26.26

Supplementary Figure 2 – Any (lower or upper) GI perforation sex- age- standardized incidence rates and adjusted contrasts between RA patients and the general population

	N	Median FU (yr)	Events	Std IR (95% CI)	Adjusted Hazard Ratios (95% CI)
GenPop	76304	4.36	374	1.22 (1.09-1.49)	Ref. 1.00
Bionaive	62524	4.97	692	1.92 (1.78-2.08)	1.13
TNFi	17594	1.19	59	1.90 (1.43-3.69)	0.97
Abatacept	2527	1.25	15	3.81 (2.03-17.1)	1.53
Rituximab	3552	2.23	23	2.10 (1.33-5.73)	1.05
Tocilizumab	2377	1.37	23	4.66 (2.80-10.5)	2.34

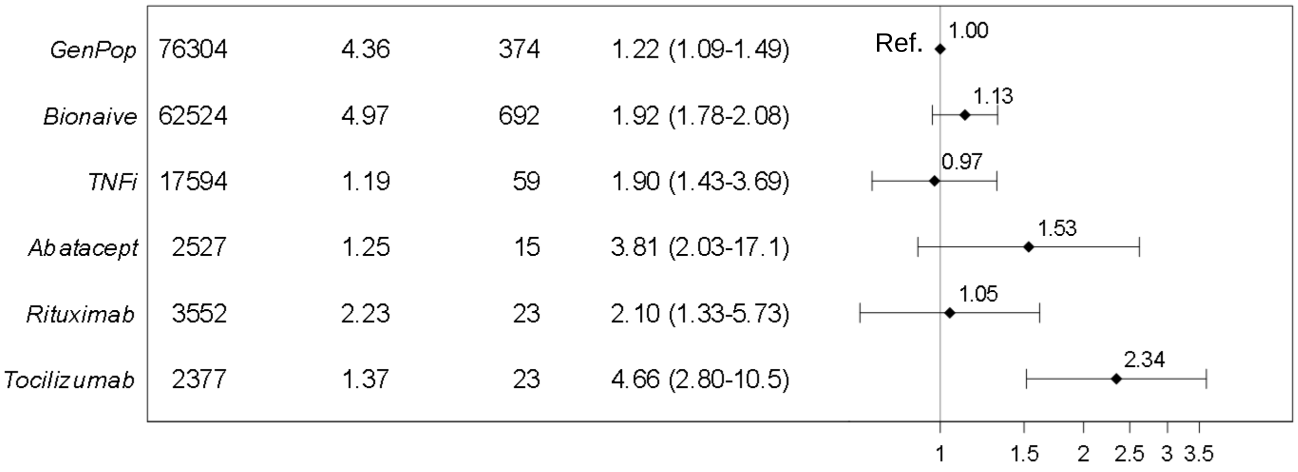

Incidence rates per 1000 person-years were standardized for sex and age (categorized in 10 years' groups) and confidence limits estimated using the method by Fay&Feuer [5].

Hazard ratios adjusted by multivariable Cox regression for: demographic characteristics (age, sex) and cumulated use of glucocorticoids. Reference group: General Population.

Abbreviations: GenPop = General Population controls; Std IR = Standardized incidence rate; FU = follow-up; yr=years.

List of References

1 Xie F, Yun H, Bernatsky S, *et al.* Brief Report: Risk of Gastrointestinal Perforation Among Rheumatoid Arthritis Patients Receiving Tofacitinib, Tocilizumab, or Other Biologic Treatments. *Arthritis & Rheumatology (Hoboken, NJ)* 2016;**68**:2612–7. doi:10.1002/art.39761

2 WHOCC - Definition and general considerations. https://www.whocc.no/ddd/definition_and_general_considera/ (accessed 30 Mar 2020).

3 Monemi S, Berber E, Sarsour K, *et al.* Incidence of Gastrointestinal Perforations in Patients with Rheumatoid Arthritis Treated with Tocilizumab from Clinical Trial, Postmarketing, and Real-World Data Sources. *Rheumatol Ther* 2016;**3**:337–52. doi:10.1007/s40744-016-0037-z

4 Curtis JR, Chen S-Y, Werther W, *et al.* Validation of ICD-9-CM codes to identify gastrointestinal perforation events in administrative claims data among hospitalized rheumatoid arthritis patients. *Pharmacoepidemiol Drug Saf* 2011;**20**:1150–8. doi:10.1002/pds.2215

5 Fay MP, Feuer EJ. Confidence intervals for directly standardized rates: a method based on the gamma distribution. *Stat Med* 1997;**16**:791–801.
